# Supplementary material for: Genomic Validation of Endometrial Cancer Patient-Derived Xenograft Models as a Preclinical Tool
Source: Int J Mol Sci. 2022 Jun 3;23(11):6266. doi: 10.3390/ijms23116266 (PMC9181722; doi:10.3390/ijms23116266)
Supplement: Supplementary file 1 [file ijms-23-06266-s001.zip › ijms-1720148-Supplementary information.pdf]

SUPPLEMENTARY INFORMATION

Genomic validation of endometrial cancer patient-derived xenograft models as a preclinical tool

Beatriz Villafranca-Magdalena, Carina Masferrer-Ferragutcasas, Carlos Lopez-Gil, Eva Coll-de la Rubia, Marta Rebull, Genis Parra, Ángel García, Armando Reques, Silvia Cabrera, Eva Colas, Antonio Gil-Moreno and Cristian P Moiola

Supplementary Tables S1 and S2

Supplementary Figures S1–S5

Table S1. Summary of the clinicopathological features of the EC patients recruited in the study.

Feature	Endometrioid	Serous
**Total of patients**	7	6
**Median Age**	64.4 (38-83)	73.3 (57-89)
**Molecular Classification**		
POLE	-	-
HCN	-	6 (100%)
LCN	-	-
MSI	7 (100%)	-
**Histologic Grade**		
Grade 1	1 (14.28%)	-
Grade 2	3 (42.86%)	-
Grade 3	3 (42.86%)	6 (100%)
**Myometrial Invasion**		
<50	4 (42.86 %)	1 (16.67%)
>50	3 (57.14 %)	4 (66.66%)
Unknown	-	1 (16.67%)
**LVSI***		
No	3 (57.14 %)	2 (33.33%)
Yes	4 (42.86 %)	3 (50%)
Unknown	-	1 (16.67%)

POLE: polymerase epsilon, HCN: high-copy number, LCN: low-copy number, MSI: microsatellite instability, LVSI: lympho-vascular space invasion.

Table S2. Description of the primary tumor areas or metastatic tissue collected for PDX development. Each patient was represented by one or more PDX models.

Characteristics of patient tumor tissue origin for PDX development			
**Patient ID**	**Area of tissue implanted**	**Number of PDXs**	**Type of tissue**
**440**	superficial	1	primary
	deep	1	primary
**505**	peritoneum	1	secondary/metastasis
**516**	superficial	1	primary
	deep	1	primary
**521**	superficial	1	primary
	deep	1	primary
**524**	superficial	1	primary
	deep	1	primary
**526**	superficial	1	primary
	deep	1	primary
**741**	superficial	1	primary
	deep	1	primary
	para-aortic lymph node	1	secondary/metastasis
**548**	superficial	1	primary
	deep	1	primary
	para-aortic lymph node	1	secondary/metastasis
**589**	peritoneum	1	secondary/metastasis
	abdominal	1	secondary/metastasis
	supramesenteric para-aortic lymph node	1	secondary/metastasis
	omentum	1	secondary/metastasis
	iliac lymph node	1	secondary/metastasis
	pelvic lymph node	1	secondary/metastasis
**596**	superficial	1	primary
	deep	1	primary
**655**	superficial	1	primary
	deep	1	primary
**782**	superficial	1	primary
	deep	1	primary
	left pelvic lymph node	1	secondary/metastasis
**003**	unspecified area	1	primary
	lymph node	1	secondary/metastasis

”

Supplementary Figure S1

a MSI:741 **b** HCN:548 **c** HCN:782

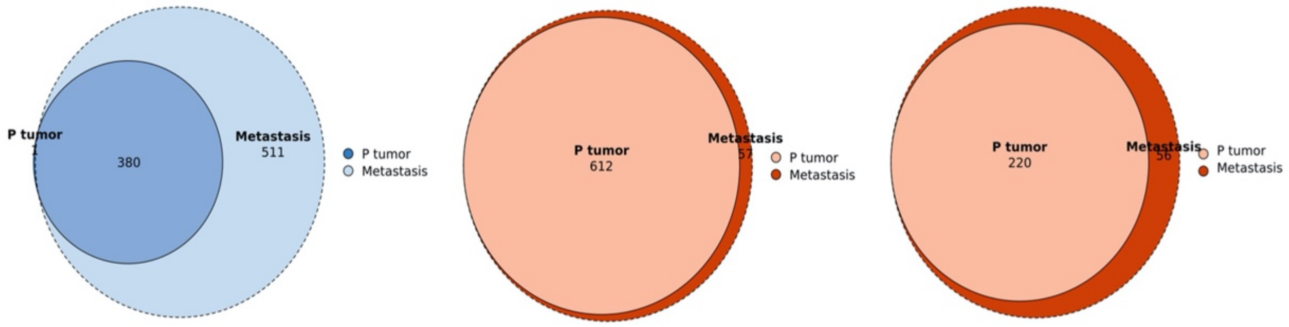

Figure S1. Comparative analysis of SNV*s* in PDX samples from primary tumor versus metastatic tissue. Venn diagram representation of three different EC PDX comparing genes carrying SNV in primary tumor versus metastatic tissue. Comparison of degree of similarity of SNVs from metastasis and primary tumor (P tumor) of a) PDX741, b) PDX548, and c) PDX782 models.

.

”

Supplementary Figure S2

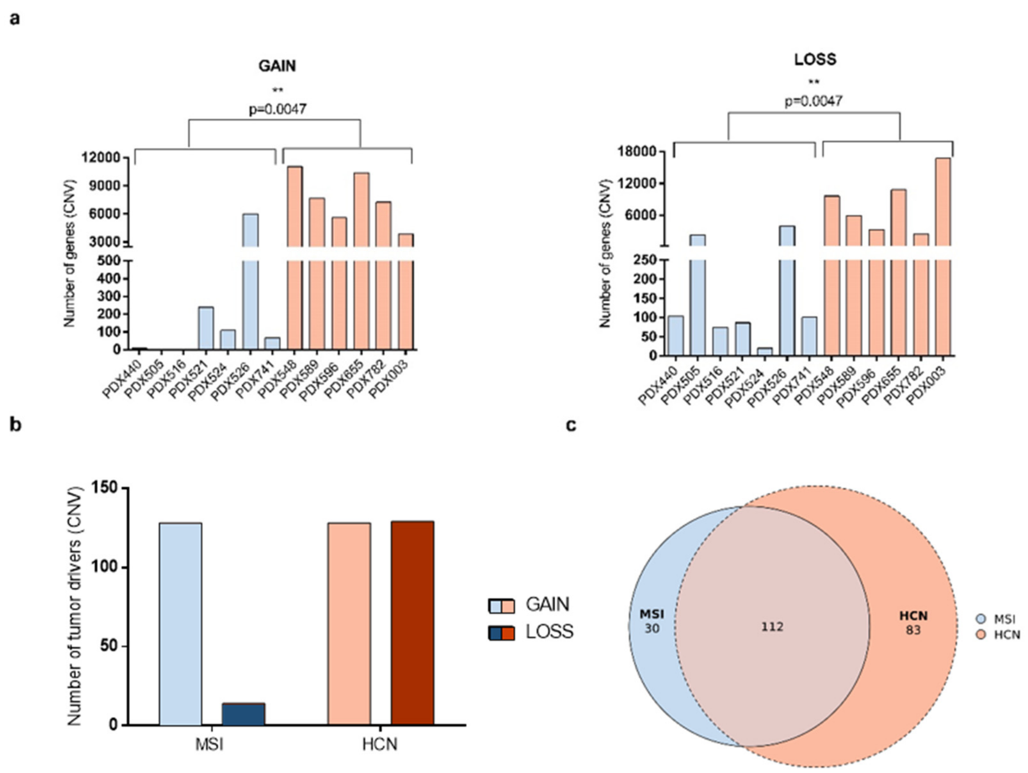

Figure S2. Molecular characterization of PDX models based on CNVs. a) Comparative analysis of the total number of genes in CNV events of GAIN (left panel) or LOSS (right panel) for each PDX model, grouped as MSI (blue bars) and HCN (orange bars). b) Analysis of the number of tumor driver genes (GAIN: amplification/LOSS: deletion) from both MSI and HCN groups present in CNV events. c) Venn diagram showing the overlap of tumor drivers genes from CNV events between MSI and HCN PDXs.

Supplementary Figure S3

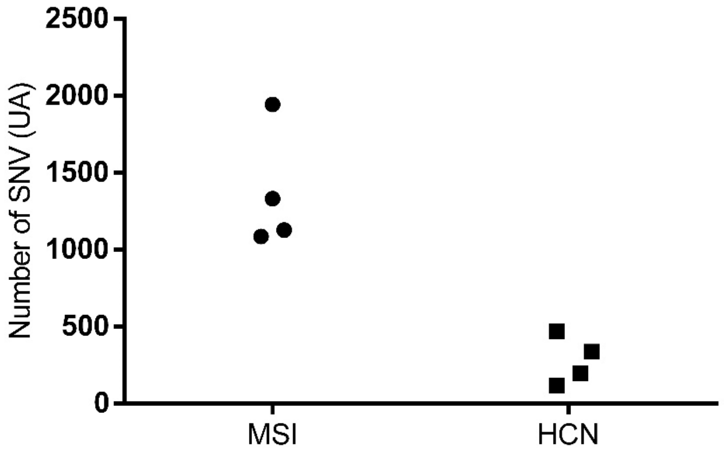

Figure S3. UA validation as surrogate sample for EC molecular characterization. Comparison of the number of genes carrying SNVs from UA liquid biopsy sample between patients classified as MSI or HCN subtypes.

Supplementary Figure S4

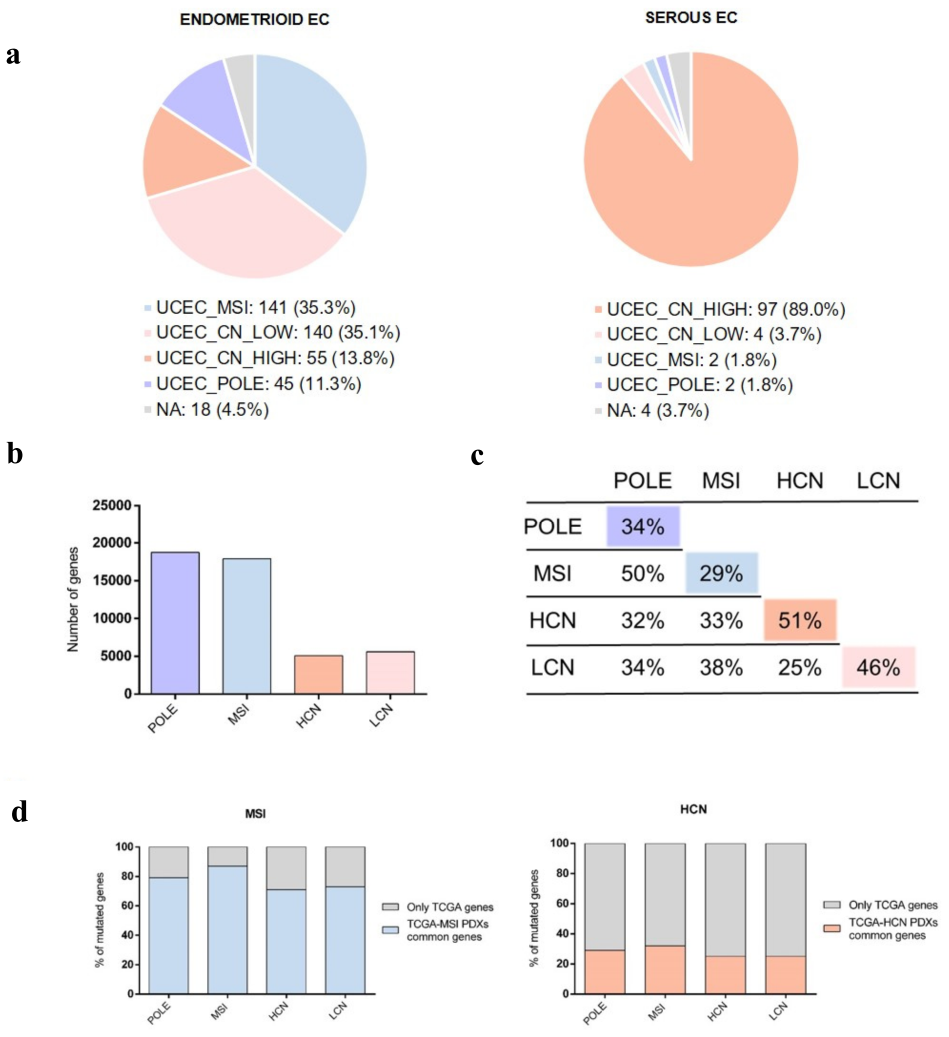

	POLE	MSI	HCN	LCN
POLE	34%			
MSI	50%	29%		
HCN	32%	33%	51%	
LCN	34%	38%	25%	46%

Figure S4. PDXs genomic validation with EC patients from TCGA database. a) Pie chart showing the number of patients classified in each EC subtype for endometrioid (left panel) or serous EC histology, obtained from TCGA PanCancer Atlas database (https://www.cbioportal.org/). Endometrioid MSI and serous HCN patients were the two subpopulations used for PDX molecular profiles comparisons. b) Total number of genes found carrying SNV in each EC subtype classification from TCGA PanCancer Atlas database. c) Table showing the percentage of genes carrying SNV considering the top 1000 most frequent altered genes for each EC molecular subtype. Intersection of the same EC molecular subtype indicates the percentage of unique genes, while intersection between two different subtypes indicate the genes shared between them. d) Comparative analysis between PDX SNV geneset to the top 1000 most frequent genes carrying SNV for each EC molecular subtype. Colored bars represent shared genes between PDX (left panel MSI PDX; right panel HCN PDX) to patients from PanCancer Atlas database, while gray bars show genes only present in patients from TCGA database.

Supplementary Figure S5

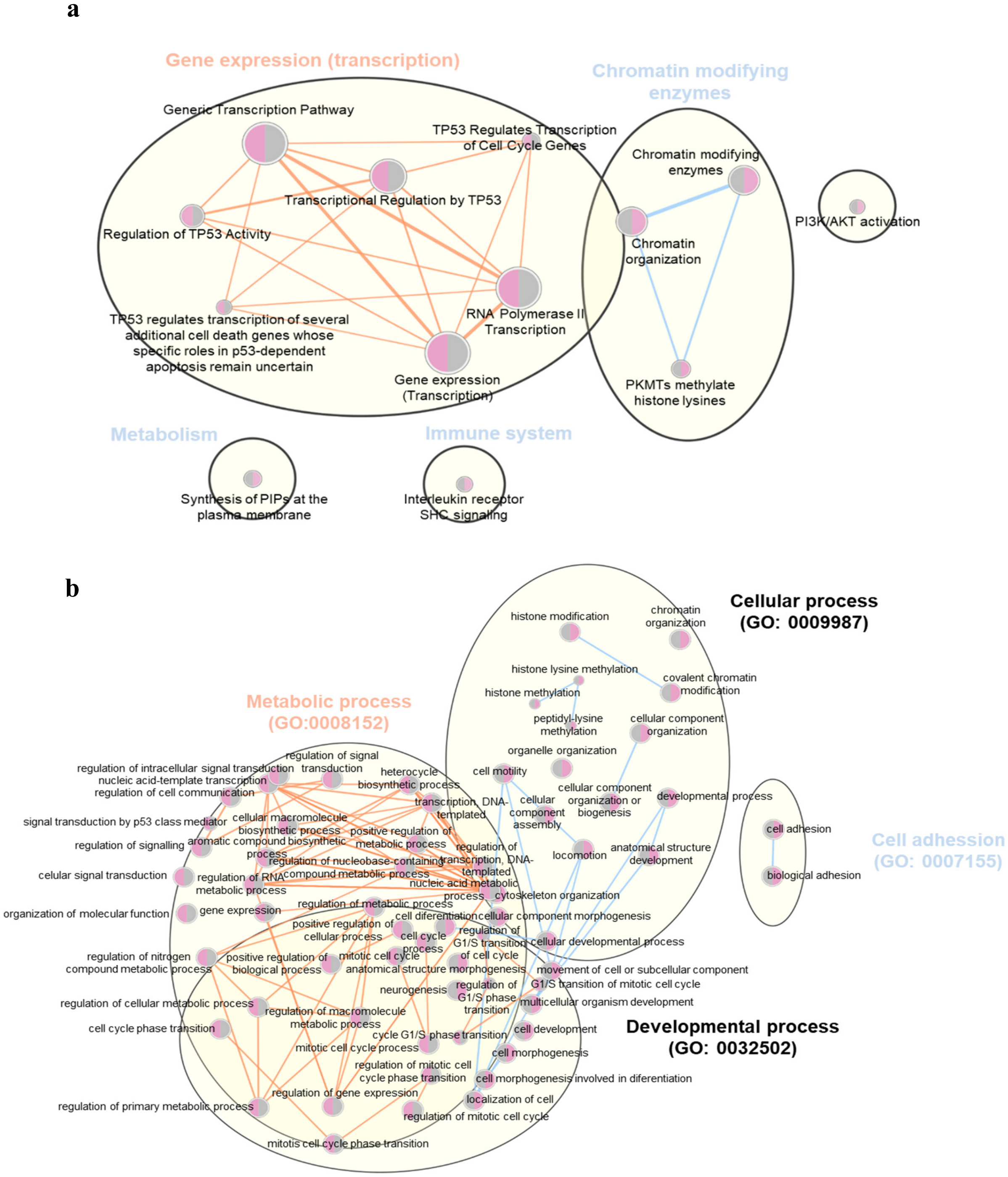

Figure S5. Functional enrichment analysis of most relevant altered genes from MSI and HCN PDX models. a) Gene ontology (BP) network of the most relevant genes associated with MSI (blue terms) or HCN (orange terms). b) Biological pathway network from REACTOME database of the most relevant altered genes in MSI (blue) and HCN PDX (orange). GO terms network analysis was performed by Cytoscape and parental GO terms were grouped using the AutoAnnotate application.
